# Conserved RNA-Binding Proteins Required for Dendrite Morphogenesis in *Caenorhabditis elegans* Sensory Neurons

**DOI:** 10.1534/g3.115.017327

**Published:** 2015-02-10

**Authors:** Simona Antonacci, Daniel Forand, Margaret Wolf, Courtney Tyus, Julia Barney, Leah Kellogg, Margo A. Simon, Genevieve Kerr, Kristen L. Wells, Serena Younes, Nathan T. Mortimer, Eugenia C. Olesnicky, Darrell J. Killian

**Affiliations:** * Department of Molecular Biology, Colorado College, Colorado Springs, Colorado 80903; † Department of Biology, University of Colorado Colorado Springs, Colorado Springs, Colorado 80918; ‡ Department of Biological Sciences, University of Denver, Denver, Colorado 80208

**Keywords:** *Caenorhabditis elegans*, dendrite morphogenesis, RNA-binding proteins, posttranscriptional regulation, PVD neurons

## Abstract

The regulation of dendritic branching is critical for sensory reception, cell−cell communication within the nervous system, learning, memory, and behavior. Defects in dendrite morphology are associated with several neurologic disorders; thus, an understanding of the molecular mechanisms that govern dendrite morphogenesis is important. Recent investigations of dendrite morphogenesis have highlighted the importance of gene regulation at the posttranscriptional level. Because RNA-binding proteins mediate many posttranscriptional mechanisms, we decided to investigate the extent to which conserved RNA-binding proteins contribute to dendrite morphogenesis across phyla. Here we identify a core set of RNA-binding proteins that are important for dendrite morphogenesis in the PVD multidendritic sensory neuron in *Caenorhabditis elegans*. Homologs of each of these genes were previously identified as important in the *Drosophila melanogaster* dendritic arborization sensory neurons. Our results suggest that RNA processing, mRNA localization, mRNA stability, and translational control are all important mechanisms that contribute to dendrite morphogenesis, and we present a conserved set of RNA-binding proteins that regulate these processes in diverse animal species. Furthermore, homologs of these genes are expressed in the human brain, suggesting that these RNA-binding proteins are candidate regulators of dendrite development in humans.

Dendrites are neuronal structures that receive sensory and synaptic information and are often elaborately branched to cast a wide receptive field or receive synaptic input from many other cells. Dendritic morphogenesis is a critical step in nervous system development, learning, and memory such that dendritic defects are associated with myriad neurologic disorders such as autism, Alzheimer disease, and schizophrenia (Jan and Jan 2010; Kulkarni and Firestein 2012). Therefore, it is important to understand the molecular genetic mechanisms that underlie dendrite morphogenesis. Although several insights into the molecular controls of dendrite morphogenesis have come from studies that have focused on transcriptional control (Parrish *et al.* 2006; Ou *et al.* 2008; Jan and Jan 2010; Iyer *et al.* 2013), there is increasing evidence that posttranscriptional mechanisms such as mRNA localization and localized translational control are important as well (reviewed by Holt and Schuman 2013). For example, the translational repressor Nanos (Nos) regulates dendrite morphogenesis and branching complexity of *Drosophila* class IV da neurons. Importantly, dendrite morphogenesis depends on proper localization of *nos* mRNA to dendrites as well as translational regulation of *nos* mRNA, both of which are mediated by *cis*-elements in the *nos* 3′ untranslated region (UTR; Brechbiel and Gavis 2008). Therefore, elucidating posttranscriptional mechanisms that regulate dendrite morphogenesis is an important research goal.

RNA-binding proteins (RBPs) are important posttranscriptional regulators of gene expression that are involved in mRNA splicing, transport, localization, stability, and translational control. Animal genomes encode a diverse suite of several hundred RBPs (Gamberi *et al.* 2006; Lee and Schedl 2006; Hogan *et al.* 2008; Kerner *et al.* 2011). Although there are some examples of mutations in RBP-encoding genes that are associated with neurologic disorders (Zhou *et al.* 2014), it is not clear how many RBPs regulate dendrite morphogenesis. Thus far, only one study has reported a systematic screen of a majority of RBP-encoding genes for function in dendrite morphogenesis. Olesnicky *et al.* (2014) reported that 63 RBP-encoding genes in the *Drosophila* genome are important for dendrite development in the larval dendritic arborization (da) neurons. However, it is not clear the extent to which RBPs involved in dendrite development are conserved across animal species. To gain insight into this question, we identified *C. elegans* homologs of the RBPs identified in the Olesnicky *et al.* (2014) study and tested each for a role in dendrite development in the worm using the multidendritic PVD neuron as a model.

The *C. elegans* PVD neuron is an excellent model for the molecular genetic investigation of dendrite morphogenesis. The bilateral PVDs, which are located between the epidermis and the body wall muscles, have extensively branched dendritic trees that function as mechanoreceptors, nociceptors, proprioceptors, and cold temperature receptors (Way and Chalfie 1989; Halevi *et al.* 2002; Tsalik and Hobert 2003; Oren-Suissa *et al.* 2010; Smith *et al.* 2010; Albeg *et al.* 2011; Chatzigeorgiou and Schafer 2011). PVD dendritic trees are stereotypic with primary (1°) branches that project anteriorly and posteriorly from the cell body and menorah- or candelabra-shaped structures extending from the primary branches, which include an orthogonal series of secondary (2°), tertiary (3°), and quaternary (4°) branches (Oren-Suissa *et al.* 2010; Smith *et al.* 2010; Figure 1A). Thus, PVD function and morphology are similar to *Drosophila* da neurons and mammalian polymodal nociceptors (Albeg *et al.* 2011).

**Figure 1:**
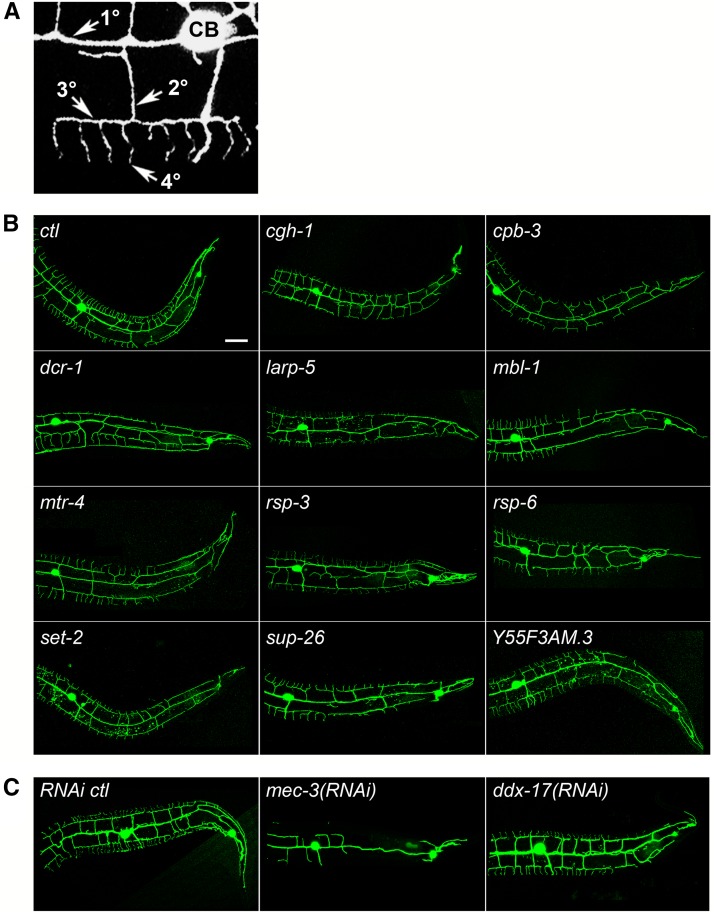
Loss or reduction of RNA-binding protein (RBP) genes results in a decrease in dendritic termini in PVD neurons. (A) PVD dendritic tree morphology includes primary (1°) branches extending from the cell body (CB) and a series of perpendicular secondary (2°), tertiary (3°), and quaternary (4°) branches. (B) Animals carrying a green fluorescent protein (GFP) marker for PVD neurons and a mutation in the RBP gene indicated have reduced dendritic termini compared with the control (*ctl*). (C) Animals carrying a GFP marker for PVD neurons and treated with RNA interference (RNAi) for the genes indicated have reduced dendritic termini compared with the control. *mec-3(RNAi)* is a positive control for RNAi and reduced dendrite phenotypes. Posterior is to the right in all images. Bar = 25 μm.

Although little is known about PVD dendrite morphogenesis, we do know that a tripartite ligand-receptor complex, which includes epidermal ligands MNR-1 and SAX-7 and the PVD receptor DMA-1, promotes PVD dendrite branching (Liu and Shen 2012; Salzberg *et al.* 2013; Dong *et al.* 2013). In addition, nascent secondary branches are stabilized by HPO-30, a PVD-expressed claudin-like transmembrane protein that likely anchors dendrites to the epidermis (Smith *et al.* 2013). We also know that the UNC-6/Netrin guidance molecule, the UNC-40/DCC receptor, and the UNC-5/Netrin receptor mediate dendrite self-avoidance (Smith *et al.* 2012). However, it is still unclear how PVD shape changes are mediated downstream of DMA-1 or Netrin signaling. Mechanical changes to dendrite morphology are, at a most basic level, the result of cytoskeleton dynamics. Interestingly, an RNA interference (RNAi) screen for PVD dendrite defects identified several genes that encode cytoskeleton-associated proteins, such as UNC-116/Kinesin-1 heavy chain, DLI-1/Dynein light intermediate chain, and BICD-1/Bicaudal-D (Aguirre-Chen *et al.* 2011). An open question is whether the DMA-1, HPO-30, Netrin, or other signaling pathways affect posttranscriptional gene regulation of genes involved in cytoskeleton dynamics, or other genes, to mediate PVD dendrite morphogenesis. Because RBPs are important posttranscriptional regulators, an investigation of the role of RBPs in dendrite morphogenesis may provide insights to this question. Thus far, no RBPs have been implicated in dendrite development in PVDs or other neurons in *C. elegans*, although a PVD-specific expression profile suggests that 47 RBP-encoding genes are enriched in expression in the PVD over other cells (Smith *et al.* 2010).

Here we identify the *C. elegans* homologs of *Drosophila* RBP genes described as important for da neuron dendrite morphogenesis (Olesnicky *et al.* 2014) and test each of them for a role in dendrite morphogenesis in PVDs. Using a combination of genetic mutations and RNAi assays, we show that reduction or elimination of function of 12 of the candidate RBP-encoding genes reveals a reduction in the number of dendritic termini in PVD neurons. Some of these genes regulate the number of secondary and tertiary dendrite branches as well. We show that each of these genes is expressed within the PVD neuron, and we use time-course analyses to show that although most of these RBPs affect terminal dendrite branch formation, two of the RBPs are required for dendrite maintenance, and one RBP is required for the timing of PVD dendrite morphogenesis. We examine the subcellular localization of each RBP within PVD neurons and discuss potential molecular roles of each RBP in this context and in the context of previous research. Because each of these RBPs functions in dendrite development in fly and worm and has at least one strong mammalian homolog, it suggests that these RBPs may be important in the development of dendrites in mammals as well. This claim is bolstered by expression data in humans showing that most of the human homologs of RBPs identified in this screen are expressed in the human brain.

## Materials and Methods

### *C. elegans* strains

Strains were derived from the Bristol strain N2, grown at 20**°**, and constructed using standard procedures (Brenner 1974). Mutants screened for dendrite defects are listed in Supporting Information, Table S1. Mutant strains obtained from the Mitani Lab through the *C. elegans* National Bioresource Project of Japan, the *C. elegans* Reverse Genetics Core Facility at the University of British Columbia, the *C. elegans* Reverse Genetics Core Facility at the Oklahoma Medical Research Foundation, and the Million Mutation Project (Thompson *et al.* 2013) were outcrossed at least four times. PVD dendrites were marked by *wdIs52[P_F49H12.4_*::*GFP]* or *wdIs51[P_F49H12.4_*::*GFP]* (Watson *et al.* 2008) for screening, *rwIs1[P_mec-7_*::*RFP]* (Smith *et al.* 2010) for gene expression studies, and *wyIs587[ser-2prom3*::*myr-mCherry]* (Liu and Shen 2012; Dong *et al.* 2013) for subcellular localization and rescue studies. The *sup*-26 expression pattern was determined using *smIs259[P_sup-26_*::*sup-26*::*GFP]* (Mapes *et al.* 2010). All other transgenes used in this study were constructed as described herein. RNAi was conducted with strain *wdIs52*; *sid-1(pk3321)*; *uIs69[P_myo-2_*::*mCherry + P_unc-119_*::*sid-1]* (Calixto *et al.* 2010).

### Imaging and quantification of PVD dendrite morphology

Worms were picked at the life stages indicated, mounted on slides with 2% agarose pads, and immobilized with 600 μM levamisole. Initial screening, time-course studies, and rescue experiments were conducted using a 40× or 63× objective on a Zeiss Axioskop or Leica DM5000B epifluorescence microscope. Dendrites, gene expression patterns, and subcellular localization were imaged with a Leica SP5 spectral confocal microscope at 63× with 0.5 μm per step and Leica LAS software. Secondary, tertiary, and terminal (quaternary and senary) dendrites were counted from the PVD cell body to the posterior end separately on the dorsal side, the ventral side, or both. Although numerous researchers contributed to the primary screen, positive hits were independently verified by confocal microscopy by a single researcher who did not participate in the primary screen. Statistical tests were performed and graphs created with Prism 6.0f software (GraphPad Software, Inc.).

### Construction of transgenes and DNA microinjection

All primers used in construction of transgenes described here are given in Table S2. For *cgh-1*, *dcr-1*, and *mtr-4*, presumptive promoter regions were amplified with polymerase chain reaction (PCR) and subcloned into the *Sph*I/*Kpn*I sites of the Fire Lab vector pPD117.01, which carries a multiple cloning site upstream of green fluorescent protein (GFP) with a *let-858* 3′ UTR. For all other genes, presumptive promoter regions, which include at least 1000 bp upstream of the start codon, or the entire upstream intragenic region, or previously published sequences, were amplified with PCR and subcloned into the pDONR221 vector using Gateway BP Clonase II (Invitrogen). Promoters were then subcloned into pDJK237, a promoterless plasmid with a Gateway cassette upstream of GFP with a 3′ UTR from *let-858* derived from pPD117.01, using Gateway LR Clonase II Plus (Invitrogen).

cDNAs were gifts of Y. Kohara or Ding Xue and James Mapes (sources given in Table S2), or were amplified from a cDNA library derived from *him-5(e1490*) created by Trizol/chloroform and first-strand synthesis by Superscript Reverse Transcriptase III (Invitrogen) and oligo dT primers. In all cases in which multiple isoforms exist, the longest isoform was selected. cDNAs were amplified with PCR without stop codons and were cloned in pDONR221 using Gateway BP Clonase II (Invitrogen). The *ser-2* promoter 3 fragment (Tsalik and Hobert 2003) was amplified with PCR and cloned into pDONR P4-P1r and the GFP coding sequence with the *let-858* 3′ UTR from pPD117.01 was amplified with PCR and cloned into pDONR P2r-P3 using Gateway BP Clonase II (Invitrogen). The cDNAs, *ser-2* promoter 3, and GFP were all cloned into pDEST R4-R3 using Gateway LR Clonase II Plus (Invitrogen).

DNA microinjection was performed using standard practices (Mello and Fire 1995). For gene expression pattern studies, *unc-76(e911)*; *rwIs1* hermaphrodites were injected with 20 ng/μL GFP plasmid and 60 ng/μL *unc-76(+)* plasmid. For subcellular localization and rescue studies, *wyIs587*; *unc-76(e911)* worms or *wyIs587*; *unc-76(e911)*; RBP mutation-bearing worms were injected with 10-20 ng/μL GFP plasmid and 60 ng/μL *unc-76(+)* plasmid.

### RNAi

RNAi feeding was performed essentially as described (Kamath and Ahringer 2003). *wdIs52*; *sid-1(pk3321)*; *uIs69[P_myo-2_*::*mCherry + P_unc-119_*::*sid-1]* L4 hermaphrodites were placed on Petri plates with nematode growth medium seeded with dsRNA-expressing *Escherichia coli*. The P_0_ worms were transferred to a new plate after 24 hr and the progeny from that second plate scored at the young adult stage. dsRNA-expressing *E. coli* were obtained from the Ahringer lab library (Fraser *et al.* 2000; Kamath *et al.* 2003) or were constructed by PCR amplification of a region of the target gene and Gateway Clonase−mediated insertion of that PCR amplicon into a double-T7 pPD129.36 plasmid modified with a Gateway cassette (Invitrogen). In cases in which target genes produce multiple isoforms, RNAi strategies targeted mRNA sequences common to all isoforms. See Table S2 for specific information on the RNAi clones used in this study.

### Blast searches

Blast searches were performed using the command line NCBI-BLAST package (version 2.2.25; Camacho *et al.* 2009). *C. elegans* protein sequences were aligned to custom BLAST databases using the BLASTp algorithm with default parameters. BLAST databases were assembled from annotated *Drosophila melanogaster* proteins (genome release 6.01, downloaded from flybase.org on 8/4/2014) and the human protein Refseq database (downloaded from NCBI on 8/4/2014) for alignments between *C. elegans* and *Drosophila* and human sequences respectively. Alignment Expect (E) values for all comparisons are reported.

### Orthology mapping and expression of human orthologs

To identify human orthologs, the *C. elegans* genes were mapped on to the Database of Orthologous Groups (OrthoDB; Waterhouse *et al.* 2013) to determine the OrthoDB identifier for each gene. All human genes assigned to the same OrthoDB identifier were taken as orthologs of the *C. elegans* gene and used for additional analysis. Data from the Tissue-specific Gene Expression and Regulation (TiGER) database (Liu *et al.* 2008) was used to assay whether the identified human orthologs are expressed in human brain tissue. Raw data files were downloaded from the TiGER website (http://bioinfo.wilmer.jhu.edu/tiger/, accessed on June 27, 2014) and analyzed using a custom Perl script.

## Results

### A screen for conserved RBPs that function in *C. elegans* PVD sensory neuron dendrite morphogenesis

Although RBPs have been shown to be important for the regulation of dendrite development (Ye *et al.* 2004; Vessey *et al.* 2008; Bestman and Cline 2008; Brechbiel and Gavis 2008; Olesnicky *et al.* 2012; Olesnicky *et al.* 2014), it is not clear the extent to which RBPs are conserved in this process across species. Thus far, only one study has aimed to identify all predicted RBP-encoding genes in a genome that contribute to dendrite morphogenesis. Olesnicky *et al.* (2014) screened the vast majority of the predicted RBP-encoding genes in the *Drosophila* genome for a role in dendrite morphogenesis of the larval class IV da sensory neurons and found that 63 of these genes were important. To determine whether these conserved RBPs function in dendrite morphogenesis in other species, we tested the homologs of these 63 RBPs for function in dendrite development using the *C. elegans* multidendritic PVD sensory neuron.

Using BLASTp, we identified 54 *C. elegans* homologs of the 63 RBPs reported to function in dendrite morphogenesis in *Drosophila* (Olesnicky *et al.* 2014). The relative smaller number of candidate RBPs in *C. elegans* is caused by several cases in which two *Drosophila* RBPs are homologous to a single *C. elegans* RBP and one case in which a *Drosophila* RBP, specifically Oskar, does not have a clear homolog in *C. elegans*. We next identified mutations and RNAi treatments to eliminate or knock down the function of each candidate RBP gene in *C. elegans*. To assay each RBP gene for a role in PVD dendrite morphogenesis, young adult mutant or RNAi-treated animals carrying a GFP marker for PVD neurons were imaged and the number of dendritic termini counted on the dorsal and ventral sides from the cell body to the posterior end of the animal (see Materials and Methods). Thirty of the 54 RBP genes were tested using genetic mutations while we used RNAi to test the remaining 24. Twenty-eight of the mutations we used are presumptive null alleles characterized by small deletions or nonsense substitutions, whereas the other two mutations were previously reported to cause a reduction of function. RNAi was used to test genes for which mutations were unavailable or to bypass pleiotropy and possibly reveal dendrite phenotypes in cases where genetic mutations resulted in embryonic or larval lethality. To increase the efficacy of RNAi in neurons, we used a neuron-sensitized knockdown strategy, which expresses the dsRNA transporter gene *sid-1* in the neurons of an otherwise *sid-1*−mutant animal (Calixto *et al.* 2010; see the section *Materials and Methods*). *mec-3(RNAi)* was used as a positive control for RNAi treatments and produced a reduced branching phenotype in PVDs similar to previously published phenotypes (Aguirre-Chen *et al.* 2011). The complete list of RBPs tested, BLASTp E values, alleles used, and RNAi treatments is given in Table S1.

**Figure 2:**
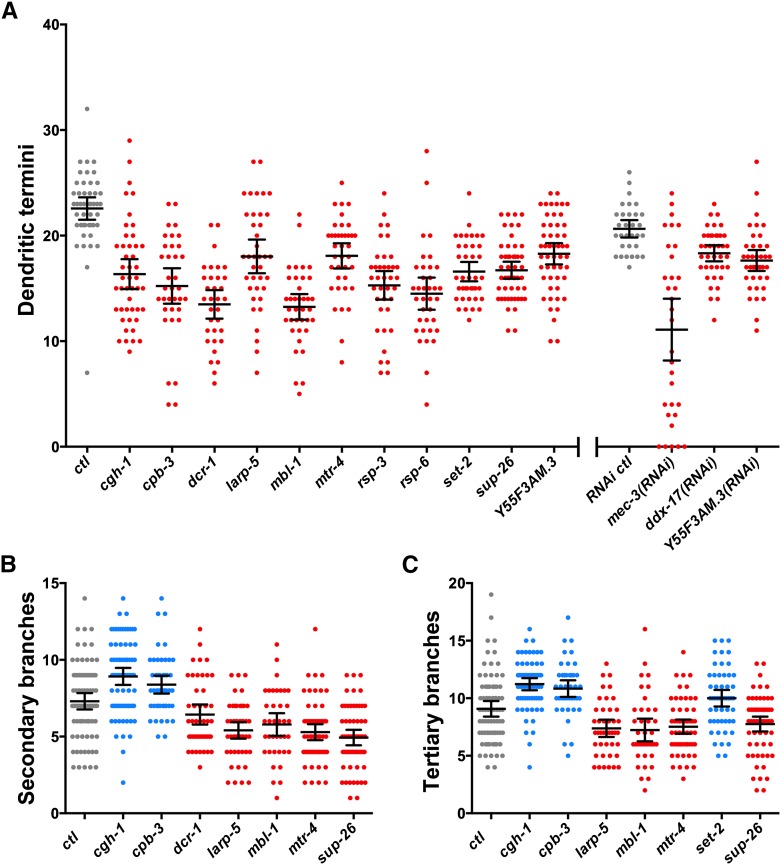
Quantification of PVD dendrite phenotypes in RNA-binding protein (RBP) mutants and RNA interference (RNAi) knockdowns. Points within each scatter column represent counts of (A) dendritic termini, (B) secondary branches, or (C) tertiary branches from the PVD cell body to the tail on the dorsal or ventral side of the worm. Lines within each column represent the means and the 95% confidence interval of the mean. Results in red and blue are significantly lower or higher respectively than controls (*ctl*) in gray based on a one-way analysis of variance test with a Fisher's Least Significant Difference multiple comparisons test with a 95% confidence interval.

We found that the loss or reduction of function of 12 RBP genes, individually, resulted in a statistically significant reduction in PVD dendritic termini compared with control animals (Table 1, Figure 1, and Figure 2A). Whereas control animals at the young adult stage have an average of 23 dendritic termini in the region scored, mutations in *cgh-1*, *cpb-3*, *dcr-1*, *larp-5*, *mbl-1*, *mtr-4*, *rsp-3*, *rsp-6*, *set-2*, and *sup-26* all result in at least a 20% reduction in dendrite termini (Figure 1B and Figure 2A). Although RNAi screening was less effective at identifying RBP genes important for dendrite morphogenesis (see the section *Discussion*), we did find that *ddx-17* and *Y55F3AM.3* RNAi-treated animals showed a 14% and 11% respective reduction in the number of dendritic termini compared to untreated control animals (Figure 1C and Figure 2A). After completing the RNAi screen, we tested several point mutations in *Y55F3AM.3* generated by the Million Mutation Project (Thompson *et al.* 2013) to identify a reduction-of-function allele. One allele, *gk454899*, affects a conserved residue across *Drosophila*, zebrafish, and mammals and resulted in a 19% reduction of dendritic termini compared to control animals (Table S1, Figure 1B, and Figure 2A).

**Table 1:** Genes identified as positive hits in a genetic screen for PVD dendrite defects

*C. elegans* RBP	*Drosophila* RBP	E Value	Predicted Protein
CGH-1	Gem3	6.00E-61	ATP-dependent DEAD-box RNA helicase
CPB-3	Orb	7.00E-64	Cytoplasmic polyadenylation element binding protein
DCR-1	Dcr-1	3.00E-120	RNase III family member; ortholog of Dicer
DDX-17	CG10777	5.00E-160	ATP-dependent DEAD-box RNA helicase
LARP-5	CG11505	8.00E-33	La-related protein with a LARP5 domain
MBL-1	Mbl	5.00E-45	CCCH zinc-finger RNA-binding domain regulator of splicing
MTR-4	L(2)35Df	0	RNA helicase; homolog of yeast Mtr4p, which is part of the TRAMP complex that is involved in various RNA processing events
RSP-3	SF2	4.00E-59	SR splicing factor required for constitutive splicing and for influencing alternative splicing
RSP-6	X16	3.00E-22	SR splicing factor required for constitutive splicing and for influencing alternative splicing; implicated in transcriptional termination
SET-2	Set1	4.00E-68	RRM domain-containing histone H3K4 methyltransferase
SUP-26	Shep	6.00E-52	RRM domain-containing protein; translational repressor
Y55F3AM.3	CG11266	1.00E-97	RRM domain-containing protein with splicing factor RBM39 linker; colocalizes with spliceosomal proteins

The *C. elegans* RBPs identified as important for dendrite morphogenesis in PVD neurons are given with an E value from a BLASTp search on *D. melanogaster* unique protein isoform database. Predicted protein function is given based on Wormbase (WS244). DEAD, Asp-Glu-Ala-Asp; CCCH, motif with three cysteine and one histidine residue; TRAMP, Trf4/Air2/Mtr4p polyadenylation complex; SR, serine/arginine rich; RRM, RNA recognition motif; H3K4, histone 3 lysine 4.

The *Y55F3AM.3* RNAi and mutant studies provide independent verification of the role of this RBP gene in PVD dendrite morphogenesis. To obtain independent verification of the role of other RBP genes in dendrite development, we obtained additional alleles where available. One additional allele of *mbl-1* and *set-2* and three additional alleles of *sup-26* confirmed a reduction of dendritic termini (data not shown). We found that *set-2(ok952)* animals had a weak reduction in the number of dendrites compared to the *n4589* allele (data not shown), which is consistent with a previous report that the *ok952* deletion is a hypomorphic allele (Xiao *et al.* 2011).

We next tested to see whether the loss or reduction of function of these 12 RBP genes also resulted in defects in lower order dendritic branching in PVDs by counting the number of secondary and tertiary branches. Loss or reduction of function of five of the 12 genes, *dcr-1*, *larp-5*, *mbl-1*, *mtr-4*, and *sup-26*, resulted in a reduction in the number of secondary branches. Interestingly, loss of function of two of the 12 genes, *cgh-1* and *cpb-3*, resulted in an increase in the number of secondary branches (Figure 2B). Loss of *cgh-1*, *cpb-3*, and *set-2* resulted in an increase of tertiary branches whereas loss of *larp-5*, *mbl-1*, *mtr-4*, and *sup-26* resulted in a reduction of tertiary branches (Figure 2C). Thus, we find that loss of *cgh-1* or *cpb-3* results in an increase of secondary and tertiary dendrite branches but also causes a roughly 30% reduction of terminal branches (Figure 2). This similarity in the *cgh-1* and *cpb-3* mutant phenotypes is interesting, given their predicted molecular functions (see the section *Discussion*). Loss of *larp-5*, *mbl-1*, *mtr-4*, or *sup-26* results in a decrease of all orders of dendrite branches (Figure 2). Notably, we did not find any mutations or RNAi treatments that resulted in supernumerary dendritic termini at the young adult stage. The pleiotropic defects in PVD neurons resulting from loss of RBP gene function are summarized in Table 2.

**Table 2:** Summary of pleiotropic defects in *C. elegans* PVD architecture resulting from loss of RBP function

RBP Gene	Reduction of Terminal Branches	3° Branch Defect	2° Branch Defect
*cgh-1*	28%	24% increase	21% increase
*cpb-3*	33%	19% increase	12% increase
*dcr-1*	40%	−	14% decrease
*ddx-17(RNAi)*	11%	−	−
*larp-5*	20%	19% decrease	28% decrease
*mbl-1*	41%	20% decrease	23% decrease
*mtr-4*	20%	17% decrease	29% decrease
*rsp-3*	32%	−	−
*rsp-6*	36%	−	−
*set-2*	27%	10% increase	−
*sup-26*	26%	15% decrease	34% decrease
*Y55F3AM.3*	19%	−	−

RBP, RNA-binding protein.

Finally, we examined all 12 RBP mutants or RNAi treatments for qualitative patterning phenotypes in the PVD. We note that *mbl-1* null mutants display a particularly striking and reproducible terminal dendrite branching defect. Although terminal branches near the cell body are similar to controls in terms of length and distribution, *mbl-1* mutants have progressively fewer and shorter terminal branches toward the posterior end of the PVD neuron (Figure 1). This finding suggests that *mbl-1* is not explicitly required for dendrite branching in general but is specifically important for the patterning of branching posterior to the cell body.

### RBPs are required for formation, timing, or maintenance of dendrite branches

A reduction in the number of terminal dendritic branches could be attributable to a failure to form the branches, a delay in branch formation relative to other hallmarks of animal development, or a failure to maintain branches. To distinguish between these possibilities, we conducted a time course analysis of dendrite development by counting dendritic termini of the PVD neuron at the mid-L4 stage, the young adult stage, and 18 hr past the young adult stage (adult).

**Figure 3:**
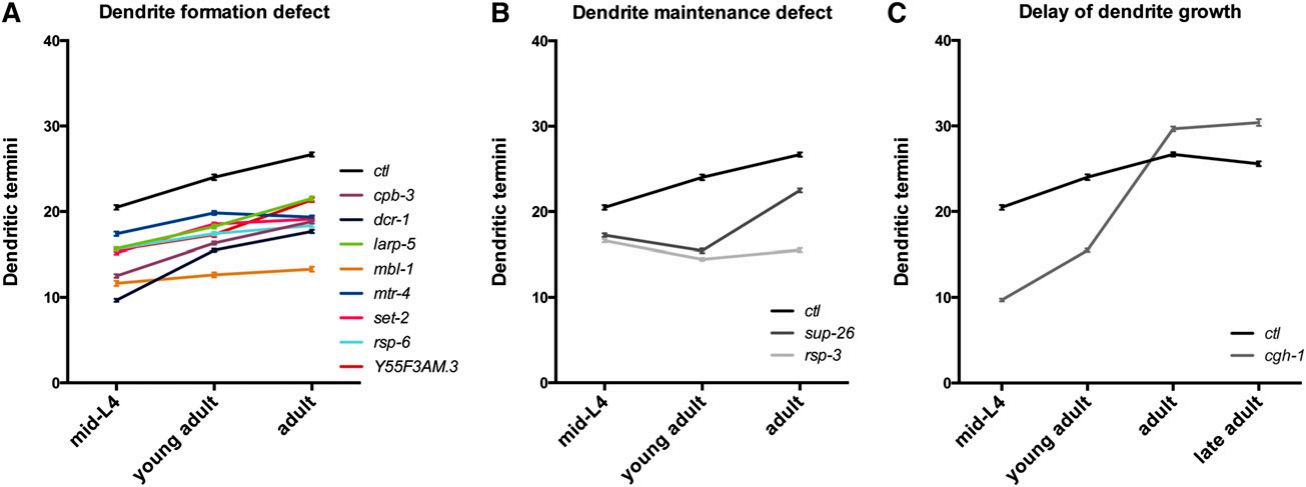
A time course analysis of dendrite development reveals defects in dendrite formation, dendrite maintenance, and timing. Control (*ctl*) or mutant animals were scored for the number of PVD dendritic termini, as before, at the mid-L4, young adult, adult (18 hr after the young adult), and sometimes late adult (48 hr after the young adult) stages. All data points for mutants are significantly different from controls based on a one-way analysis of variance (ANOVA) test with a Fisher’s Least Significant Difference multiple comparisons test with a 95% confidence interval. Data points are the mean values where *n* = 80 for each genotype. Error bars show the standard error of the mean. (A) Mutants indicated have a dendrite formation defect. (B) Mutants indicated have a dendrite maintenance defect in the L4 stage. Mutant values at each time point are significantly different from each other time point based on a one-way ANOVA test with a Fisher's Least Significant Difference multiple comparisons test with a 95% confidence interval. (C) *cgh-1* mutants have a delay in dendritic termini formation.

We found a statistically significant reduction of dendritic termini in *cpb-3*, *dcr-1*, *larp-5*, *mbl-1*, *mtr-4*, *set-2*, *rsp-6*, and *Y55F3AM.3* mutants relative to the control at all stages (Figure 3A). This finding suggests that these mutants fail to form the appropriate number of terminal branches and that there is neither a delay nor a maintenance defect.

The time course analyses suggest that *sup-26* and *rsp-3* are required for dendrite maintenance during the L4 stage. We found that young adult *sup-26* mutant animals have a statistically significant, roughly 10% reduction in dendritic termini compared to mid-L4 stage mutant animals (Figure 3B). The number of dendrite termini in *sup-26* mutants then increases significantly during the adult stage but does not reach the same level as controls (Figure 3B). Similar to *sup-26* mutants, *rsp-3* mutants exhibit a statistically significant loss of roughly 9% of dendritic termini in PVD neurons from the mid-L4 to the young adult stage (Figure 3B). Adult *rsp-3* mutant animals exhibit a mild but statistically significant increase in the number of dendrites from the young adult stage but never recover beyond the number of dendritic termini observed in mid-L4 animals (Figure 3B). Together, this finding suggests that *sup-26* and *rsp-3* mutants lose more terminal dendrite branches than they form during the late-L4 stage. However, branch loss ceases during the adult and some growth ensues, albeit weakly in *rsp-3* mutants. We also find that *sup-26* and *rsp-3* mutants have significantly fewer dendrites than controls at the mid-L4 stage (Figure 3B) shortly after fourth order dendrite branch outgrowth is initiated in the early L4 stage (Smith *et al.* 2010). This finding suggests that *sup-26* and *rsp-3* mutants are defective in maintenance of terminal branches as soon as they begin to develop or that these mutants fail to form the appropriate number of dendritic termini initially.

Interestingly, we find that *cgh-1* mutants are delayed in dendrite development with a 51% reduction at the mid-L4 stage but only a 35% reduction at the young adult stage. As adults and late adults (48 hr past the young adult stage), there is no longer a reduction in dendritic termini in *cgh-1* mutants relative to controls. In fact, *cgh-1* mutants have statistically significantly more terminal branches than controls at these later stages (Figure 3C). Collectively, the time course analyses demonstrate at least three different etiologies for dendrite defects; hence, the RBPs identified in this study likely employ different molecular mechanisms for dendrite morphogenesis.

### RBP genes required for dendrite development are expressed in the PVD neuron

**Figure 4:**
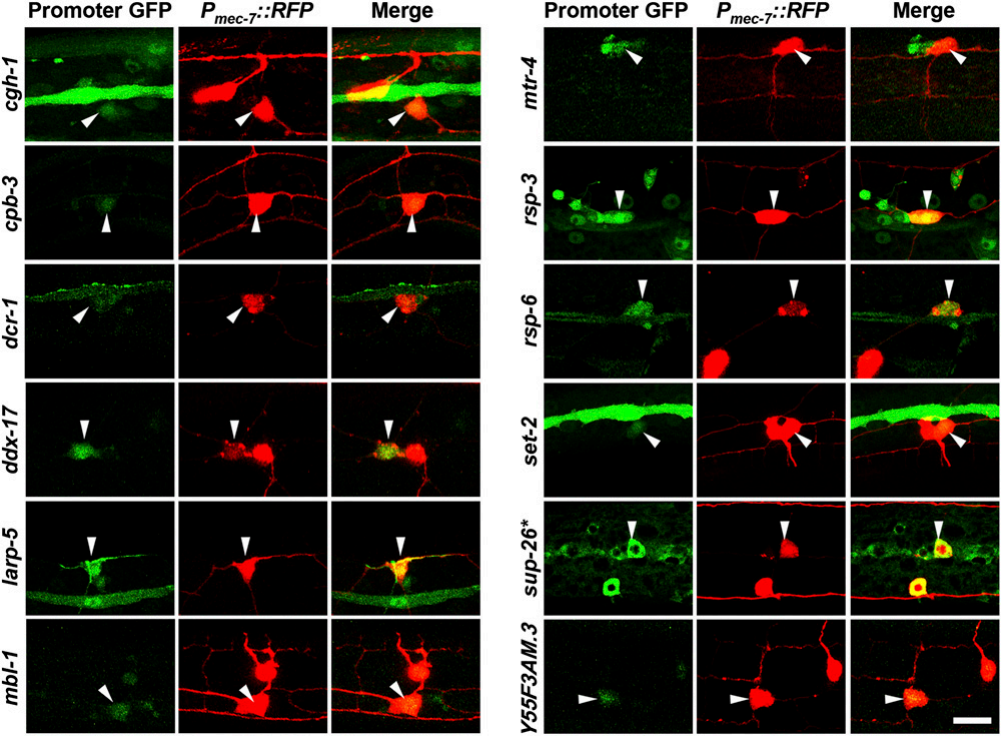
RNA-binding protein (RBP) genes that are important for PVD dendrite morphogenesis are expressed in the PVD neuron. Presumptive promoter regions for each RBP gene indicated were fused to green fluorescent protein (GFP) to determine whether they are expressed in PVD neurons, which were marked with P*_mec-7_*::*RFP*. Arrowheads mark the PVD cell body. *sup-26 expression was determined from a *P_sup-26_*::*sup-26 cDNA*::*GFP* construct. Bar = 10μm.

To determine the expression patterns for each of the RBP genes identified in our screen, we expressed the coding sequence of GFP under the control of presumptive promoters for each RBP gene (see the section *Materials and Methods*). Each of the 12 RBP gene regulatory regions expressed GFP in the PVD neuron (Figure 4). Most of the genes (*cgh-1*, *cpb-3*, *dcr-1*, *mtr-4*, *rsp-3*, *rsp-6*, *set-2*, and *sup-26*) are expressed broadly throughout development (data not shown) excluding the germ line, which often silences repetitive DNA such as the extrachromosomal arrays generated by DNA microinjection in worms (Mello *et al.* 1991; Kelly and Fire 1998). In contrast, *ddx-17*, *larp-5*, *mbl-1*, and *Y55F3AM.3* are expressed mostly or exclusively in neurons including the PVD (data not shown). These data demonstrate that RBP genes required for PVD dendrite development are expressed in the PVD, and our findings are consistent with a cell-autonomous function of the RBPs within the PVD neuron. However, RBP gene expression is not specifically restricted to the PVD neuron, suggesting that these RBP genes may play additional roles in other neurons and, in some cases, non-neural tissues.

### Subcellular localization of RBPs suggests how they influence dendrite development

Because RBPs play diverse molecular roles related to posttranscriptional gene regulation, the subcellular localization of any given RBP may offer some insight as to which role(s) it does or does not play. To determine where within the PVD each RBP is localized, we expressed each RBP as a fusion to GFP in PVD neurons using the *ser-2prom3* regulatory element (Tsalik and Hobert 2003; see the section *Materials and Methods*).

**Figure 5:**
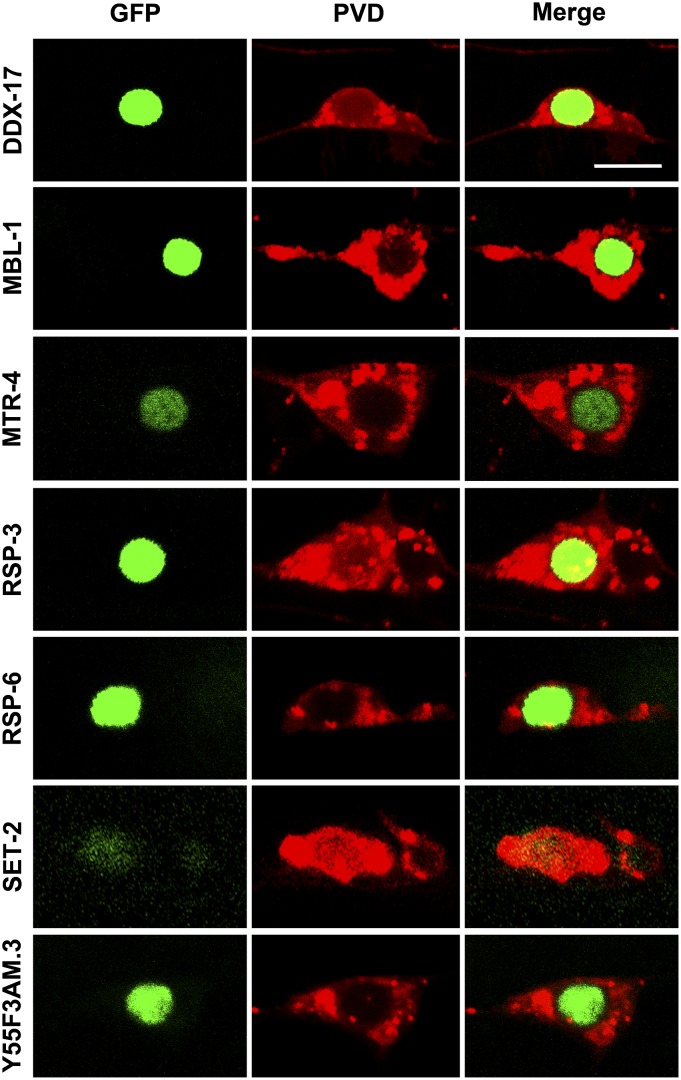
Nuclear localization of several RNA-binding proteins (RBPs) in PVD neurons. cDNAs for RBP genes indicated were fused to green fluorescent protein (GFP) under the control of a PVD-specific promoter and reveal a nuclear localization. PVDs were marked by *ser-2prom3*::*myr-mCherry*. Bar = 5 μm.

**Figure 6:**
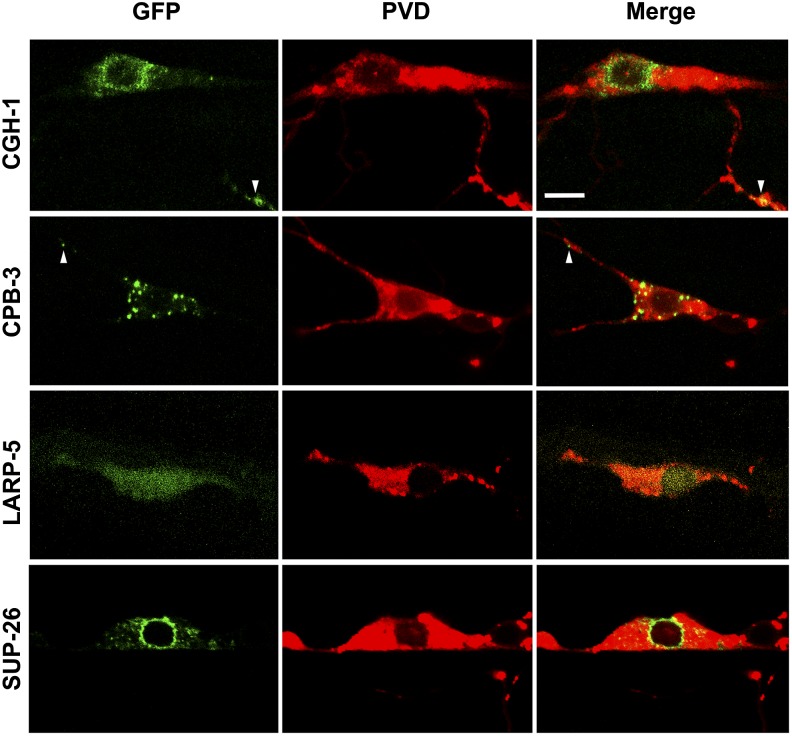
Cytoplasmic localization of CGH-1, CPB-3, LARP-5, and SUP-26 in PVD neurons. cDNAs for RNA-binding protein (RBP) genes indicated were fused to green fluorescent protein (GFP) under the control of a PVD-specific promoter and reveal cytoplasmic localization. PVDs were marked by *ser-2prom3*::*myr-mCherry*. Arrowheads indicate GFP-positive particles within dendrites. Bar = 5 μm.

DDX-17, MBL-1, MTR-4, RSP-3, RSP-6, SET-2, and Y55F3AM.3 translation fusions to GFP show clear nuclear expression (Figure 5). This finding is consistent with these RBPs playing roles in RNA processing within the nucleus (see the section *Discussion*). Conversely, we find that CGH-1, CPB-3, LARP-5, and SUP-26 translational fusions to GFP are localized to the cytoplasm (Figure 6). More specifically, CGH-1::GFP is enriched in the perinuclear region and small puncta are present throughout the cytoplasm, including particles in dendrites (Figure 6). Similarly, CPB-3::GFP also is found in puncta in the cytoplasm of the cell body and dendrites. However, CPB-3 puncta are substantially larger and lack perinuclear enrichment (Figure 6). Unlike CGH-1 and CPB-3, SUP-26::GFP is restricted to the cytoplasm of the cell body and is not found in dendrites. SUP-26 has a striking, perinuclear enrichment with intermediate-sized puncta, relative to CGH-1 and CPB-3, located further from the nucleus but still within the cell body (Figure 6). Finally, LARP-5::GFP is diffuse throughout the cytoplasm, including the dendrites, and the nucleus and does not localize to discrete puncta (Figure 6).

We were not able to generate a visible DCR-1::GFP fusion protein expressed in the PVD neuron. However, anti-DCR-1 immunohistochemistry reveals that DCR-1 is in puncta in the cytoplasm and nucleus of *C. elegans* germ cells (Beshore *et al.* 2011). This finding is consistent with known roles of DCR-1/Dicer in the microRNA and RNAi pathways as well as restricting the accumulation of dsRNA from bidirectional transcription (Bernstein *et al.* 2001; Grishok *et al.* 2001; White *et al.* 2014).

**Figure 7:**
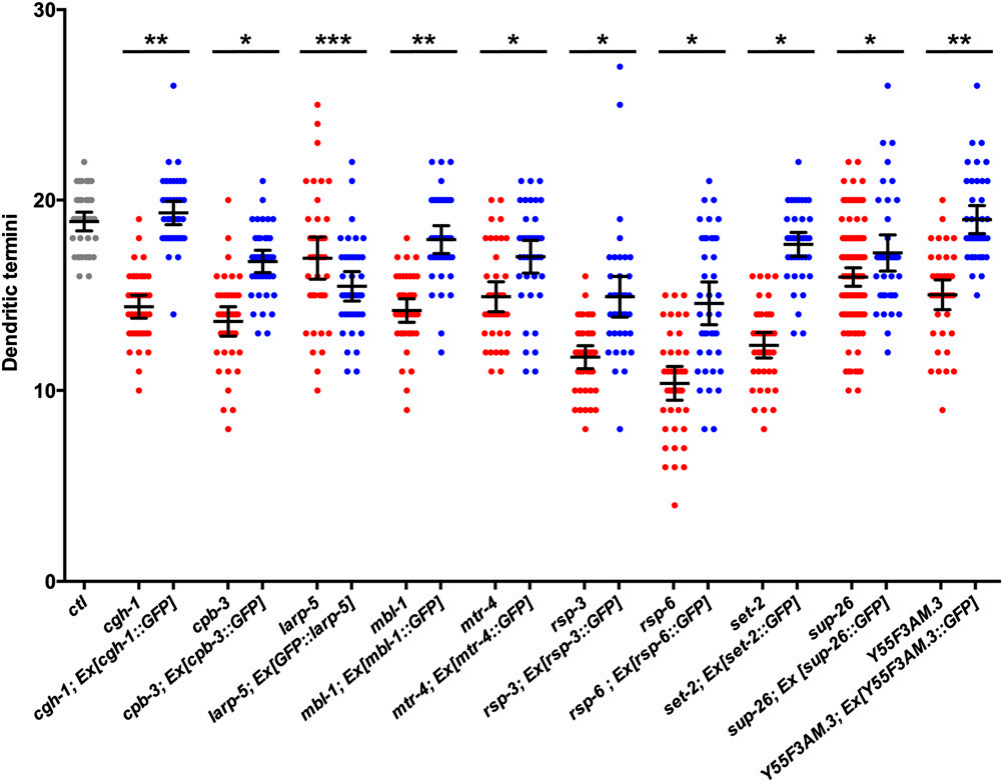
PVD-specific expression of RNA-binding protein::green fluorescent protein (RBP::GFP) fusion proteins rescues dendrite defects in most of the RBP mutants. Control (*ctl*) animals (gray), mutant animals (red), and mutant animals bearing an extrachromosomal array (Ex) that expresses the corresponding RBP::GFP fusion protein in the PVD neuron (blue) were scored for the number of dendritic termini using the *ser-2prom3*::*myr-mCherry* PVD marker. Points within each scatter column represent counts of dendritic termini from the PVD cell body to the tail on the dorsal or ventral side of the worm. Lines represent the means and the 95% confidence interval of the mean. All of the mutants are significantly lower than the control. *Mutants expressing RBP::GFP are significantly different from mutants and significantly different from controls indicating a partial rescue. **Mutants with RBP::GFP are significantly different from mutants and not significantly different from controls indicating a complete rescue. ***GFP::LARP-5 causes a significant reduction in dendritic termini compared to the *larp-5* mutant alone. All statistics are based on a one-way ANOVA test with a Fisher’s Least Significant Difference multiple comparisons test with a 95% confidence interval.

To test whether these RBPs function cell-autonomously in the PVD neuron and to determine if RBP::GFP fusion protein subcellular localization is biologically relevant, we expressed each RBP::GFP transgene specifically in the PVD neuron and tested for its ability to rescue PVD dendrite defects in RBP mutant animals (see the section *Materials and Methods*). We found that PVD-specific expression of CGH-1, MBL-1, and Y55F3AM.3 fusion proteins to GFP confer a statistically significant complete rescue of the PVD dendritic termini defects in the respective mutants (Figure 7). This strongly suggests that CGH-1, MBL-1, and Y55F3AM.3 each function cell-autonomously in the PVD to control dendrite development. Furthermore, the subcellular localizations inferred from these RBP::GFP fusions proteins are likely accurate because these proteins are functional.

We also found that *cpb-3*, *mtr-4*, *rsp-3*, *rsp-6*, *set-2*, and *sup-26* dendrite defects are rescued partially by PVD-specific expression of the corresponding RBP::GFP fusion proteins. The reduction of PVD dendritic termini associated with each mutant is significantly ameliorated by expression of the corresponding RBP::GFP fusion protein, but does not return to control levels (Figure 7). This finding suggests that these RBPs function within the PVD neuron to control dendrite development. However, the partial rescue does not allow us to exclude the possibility that RBP expression in other cells is also important for PVD dendrite development. Alternatively, partial rescue may be the result of GFP slightly reducing the activity of the RBPs or non-native expression levels of the RBP::GFP fusion proteins.

We were unable to recover transgenic lines expressing LARP-5::GFP, suggesting that this transgene is toxic. However, we were able to generate transgenic animals expressing GFP::LARP-5. Interestingly, we found that *larp-5* mutants expressing a GFP::LARP-5 fusion protein in PVD neurons were not rescued; rather, these animals had significantly lower numbers of dendritic termini than *larp-5* mutants alone (Figure 7). It is possible that a GFP tag on LARP-5 disrupts its function. Alternatively, the results may indicate that LARP-5 levels must be precisely controlled for proper dendritic development. This would not be unusual given that the expression levels of multiple RBPs are critical for *Drosophila* da sensory neuron development. For example, overexpression of *nanos*, *pumilio*, and *brat* all result in more severe dendrite defects than the mutants alone (Ye *et al.* 2004; Olesnicky *et al.* 2012). We were unable to test DDX-17::GFP for cell-autonomous function and relevance of its subcellular localization since this RBP was identified from RNAi, and *ddx-17* mutants die early in development.

## Discussion

### Conservation of the role of RBPs in dendrite morphogenesis

Sixty-three RBP genes were reported to function in *Drosophila* da neuron morphogenesis, suggesting that many aspects of posttranscriptional regulation are important to this process (Olesnicky *et al.* 2014). Here, we test 54 homologs of the *Drosophila* suite of dendrite RBPs and show that 12 of them, or roughly 22%, are important for PVD dendrite morphogenesis. This relatively low rate of functional conservation may not be unexpected. Only 21% of *C. elegans* genes with an ortholog in another eukaryote give obvious RNAi phenotypes (Kamath *et al.* 2003). In addition, the plasticity of genetic networks between *C. elegans* and *C. briggsae*, which share similar body plans and ecological niches despite roughly 20 million years of evolution, is significant; over 25% of orthologs in these worm species have different functions (Verster *et al.* 2014).

There are few reasons why such a small percentage of the RBP genes required for *Drosophila* da neuron morphogenesis have homologs with conserved function in *C. elegans* PVD neurons. Although da neurons and PVD neurons are both complex multidendritic sensory neurons, the PVD has a simpler and more stereotyped shape, which may not require as many posttranscriptional regulatory factors. Another significant difference between da neurons and PVDs is that da neurons undergo extensive pruning and shape changes during development while the PVD neuron does not undergo drastic morphological changes (Williams and Truman 2005a,b; Albeg *et al.* 2011). Perhaps the reduced need for shape changes in PVDs also reduces the need for posttranscriptional gene regulation mechanisms mediated by RBPs.

Although in this study we specifically tested 54 RBP genes using predominantly predicted null alleles and a neuron-sensitized RNAi strategy, the 12 genes reported are likely an underestimate. Although 10 of 30 genetic mutations tested positive for dendrite defects, our primary RNAi screen identified only two (*ddx-17* and *Y55F3AM.3*) of 24 positive hits. On the basis of the rate of positive hits using genetic mutations, we would have expected a greater percentage of the genes tested by RNAi to also be positive. Moreover, after completing the RNAi screen, we identified some mutations from the Million Mutation Project (Thompson *et al.* 2013) that affect genes for which no deletion allele was available. Using these alleles, we confirmed *Y55F3AM.3* and added *larp-5*, which was not a hit in the initial RNAi screen. Thus, we conclude that false negatives by RNAi are likely and difficult to avoid. Some of the genes tested by genetic null mutation also may be reported as false negatives in our screen due to maternal rescue in cases where homozygous sterile mutants are scored from heterozygous mothers (see below for discussion of *drsh-1/Drosha*). Finally, although most alleles tested are predicted nulls, at least two have been reported to be hypomorphic alleles. Homologs of *stau-1* are involved in dendrite development in various species, yet we did not find statistically significant defects in PVD neurons using the hypomorphic allele *tm2266* (Tang *et al.* 2001; Barbee *et al.* 2006; Goetze *et al.* 2006; Legendre *et al.* 2013). The *sym-2(mn617)* allele is also likely to be a hypomorph (Yochem *et al.* 2004) and did not produce PVD defects in our screen. Although the *sym-2* homolog in *Drosophila*, *glo*, is required for dendrite development (Brechbiel and Gavis 2008; Olesnicky *et al.* 2014), this has not been demonstrated for the human homolog, heterogeneous nuclear ribonucleoprotein F.

Although the dendritic roles of several RBP genes identified in the Olesnicky *et al.* (2014) RNAi screen were confirmed by genetic mutation or second RNAi line, it is possible that some false positives were reported because of a previously unknown problem with some RNAi fly stocks used. A recent study showed that many randomly selected Vienna *Drosophila* RNAi Center “KK” series RNAi stocks produce nonspecific phenotypes when crossed to certain Gal4 drivers due to an additional and previously uncharacterized RNAi hairpin vector insertion site (Green *et al.* 2014). We therefore acknowledge this caveat to our assessment of the number of conserved RBPs that function in dendrite morphogenesis in both *Drosophila* da neurons and *C. elegans* PVDs. Considering the results of the *Drosophila* da neuron dendrite screen and this study, the percent of RBPs that play a conserved role in dendrite morphogenesis is likely to exceed the 22% reported here.

### mRNA localization and translational regulation contribute to dendrite morphogenesis

It is well documented that mRNAs are transported within RNA−protein complexes (RNPs) into dendrites, and there is increasing evidence that translational control of these mRNAs contributes to dendrite morphology (reviewed by Bramham and Wells 2007; Pimentel and Boccaccio 2014; Tom Dieck *et al.* 2014; Di Liegro *et al.* 2014). Therefore, we were not surprised that this study identified RBP genes, such as *cgh-1*, *cpb-3*, *larp-5*, and *sup-26*, which are implicated in mRNA transport, localization, and/or translation.

*cgh-1* encodes an RNA helicase similar to mammalian DDX6/RCK/p54. Both are associated with various RNP granules and are required for mRNA translational repression, possibly by stalling translation by polysomes associated with RNPs (reviewed by Rajyaguru and Parker 2009; Pimentel and Boccaccio 2014). We found that functional CGH-1::GFP protein is enriched in the cell body and in puncta within PVD dendrites (Figure 6 and Figure 7) suggesting that CGH-1 may act as a translational repressor of target mRNAs during their transport into dendritic compartments.

*cpb-3* encodes a homolog of mammalian cytoplasmic polyadenylation element binding protein (CPEB), which acts as a translational repressor of target mRNAs but also promotes translation when phosphorylated (reviewed by Villalba *et al.* 2011). Although invertebrate CPEBs, such as CPB-3, lack the site of phosphorylation (Hasegawa *et al.* 2006), it is possible that their dual-function as a repressor and activator may be regulated by an alternative mechanism. CPEB has known roles within the nervous system where it is required for learning and memory by regulating synaptic plasticity (Si *et al.* 2003; Miniaci *et al.* 2008). In addition, CPEB regulates the transport of mRNAs in dendrites and disruption of CPEB function in frogs led to stunted development of dendritic arbors (Huang *et al.* 2003; Bestman and Cline 2008). Because CPEB-containing granules are found within dendrites in frogs and a functional CPB-3::GFP protein is found within dendrites in *C. elegans* (Bestman and Cline 2009; Figure 6 and Figure 7), it suggests that CPEB-dependent translation is important for providing localized sources of proteins within the dendritic compartment, which in turn regulates local dendrite development.

*sup-26* encodes an RNA recognition motif-containing protein similar to *Drosophila* Shep and mammalian RBMS1/2/3. *C. elegans* SUP-26 has been shown to directly bind to the 3′ UTR of a target mRNA and repress its translation, possibly through a physical interaction with poly(A)-binding protein 1 (Mapes *et al.* 2010). *Drosophila shep* was shown recently to be important for neuronal remodeling (Chen *et al.* 2014), suggesting a broader role in nervous system development. A functional SUP-26::GFP fusion protein is largely perinuclear and absent from dendrites (Figure 6 and Figure 7) and thus may play a role in repressing the translation of target mRNAs upon nuclear export and before they associate with RNPs that take over the role of mRNA localization and translational control.

LARP-5 is homologous to mammalian LARP4 and LARP4B, which encode proteins that bind to the poly(A)-binding protein, associate with polysomes, and promote mRNA stability and translation (Schäffler *et al.* 2010; Yang *et al.* 2011). siRNA-mediated knockdown of LARP4 leads to decreased mRNA levels, whereas overexpression of LARP4 increases mRNA levels, strongly suggesting that LARP4 can prolong mRNA half-life (Yang *et al.* 2011). In *C*. *elegans*, LARP-5::GFP is localized to the cytoplasm including the dendrites (Figure 6), which is consistent with association with poly(A)-binding protein and polysomes and suggests that LARP-5 may promote the stability of target mRNAs that are required for dendrite development. However, the subcellular localization of LARP-5 must be considered in context of the caveat that GFP::LARP-5 does not rescue dendrite defects of *larp-5* mutants and actually leads to a significant further reduction of dendritic termini. Thus the subcellular localization we observe may not reflect endogenous LARP-5 protein localization (Figure 7).

Taken together, we favor a model whereby SUP-26 translationally represses mRNAs upon nuclear export and before loading with additional RBPs, possibly including CGH-1, CPB-3, and/or LARP-5, which regulate mRNA transport, stability, and translation. In the absence of SUP-26, target mRNAs may be prematurely translated in the cell body where they cannot influence dendrite development. In support of this idea, *sup-26* mutants have fewer branches at all orders (Figure 2). In the absence of CGH-1 and CPB-3, target mRNAs may be translated prematurely within dendrites, which could plausibly explain the excess secondary and tertiary branches observed in *cgh-1* and *cpb-3* mutant PVDs (Figure 2). Terminal branches eventually do form in *cgh*-1 mutants (Figure 3C), suggesting that over time target mRNAs or their protein products do eventually reach the correct location or exhibit the correct activity. Finally, we suggest that a loss of LARP-5 activity results in decreased mRNA stability and thus a reduction of branching at all orders (Figure 2). These findings highlight the importance of mRNA localization, stability, translational repression, and localized protein synthesis within the developing dendritic arbor.

### The role of mRNA splicing in dendrite morphogenesis

One striking finding of this screen is that six (MBL-1, RSP-3, RSP-6, SET-2, Y55F3AM.3, and DDX-17) of the 12 RBPs that we identified as important for PVD dendrite morphogenesis are known or thought to be involved in mRNA splicing, and more specifically alternative splicing.

*mbl-1* encodes a homolog of *Drosophila* Muscleblind (Mbl) and mammalian Muscleblind-like proteins (Mbnl), which are Zinc finger containing proteins that regulate alternative splicing (Begemann *et al.* 1997; Kanadia *et al.* 2003; Ho *et al.* 2004; Pascual *et al.* 2006; Wang *et al.* 2008; Sasagawa *et al.* 2009; Spilker *et al.* 2012; Wang *et al.* 2012). Although *C. elegans* MBL-1 has not been shown definitively to participate in alternative splicing, strong homology to fly Mbl and mammalian Mbnl proteins strongly suggests that this molecular function is conserved (Pascual *et al.* 2006; Spilker *et al.* 2012).

*rsp-3* and *rsp-6* provide another strong link to alternative splicing. These genes encode members of the serine/arginine-rich (SR) family of splicing factors that regulate constitutive and alternative splicing (reviewed by Risso *et al.* 2012). *rsp-3* encodes a protein similar to *Drosophila* SF2 and human SRSF1/9, and *rsp-6* encodes a homolog of *Drosophila* X16 and mammalian SRSF3/7 splicing factors (Krainer *et al.* 1991; Kawano *et al.* 2000; Longman *et al.* 2000; Vorbrüggen *et al.* 2000; De La Mata and Kornblihtt 2006).

Although little is known about *Y55F3AM.3* and *ddx-17*, the paucity of information does suggest that these genes may be involved in alternative splicing. Y55F3AM.3 is an RRM-containing protein with a human homolog (RBM39) that co-localizes with spliceosomal proteins and affects alternative splicing for some target genes (Imai *et al.* 1993; Dowhan *et al.* 2005; McCracken *et al.* 2005; Ellis *et al.* 2008; Huang *et al.* 2012). *ddx-17* encodes a DEAD-box RNA helicase protein similar to human DDX17/p72 (and DDX5/p68), which is a regulator of alternative splicing and co-purifies with the U1snRNP and SR protein SRrp86 (Hönig *et al.* 2002; Lee 2002; Li *et al.* 2003). Furthermore, one study suggests that DDX5/p68 facilitates the binding of Mbnl proteins to splicing targets (Laurent *et al.* 2012), adding another link to alternative splicing.

Finally, *set-2* encodes an RRM-containing histone 3 lysine 4 (H3K4) methyltransferase similar to yeast and fly Set1 and human SETD1A and SETD1B (Xu and Strome 2001; Briggs *et al.* 2001; Miller *et al.* 2001; Lee and Skalnik 2005; Xiao *et al.* 2011; Table 1). Set1 is recruited by RNA polymerase II to mediate H3K4 trimethylation (me3) on nearby nucleosomes. Thus, H3K4me3 is a landmark of actively transcribed genes or genes that experience transcription initiation (Miller *et al.* 2001; Krogan *et al.* 2003; Tenney and Shilatifard 2005; Guenther *et al.* 2007). In addition, H3K4me3 enhances splicing efficiency and affects alternative splicing, possibly through its association with the U2 snRNP (Sims *et al.* 2007; Luco *et al.* 2010). Furthermore, H3K4me3 is enriched at alternative transcriptional start sites suggesting it may regulate alternative promoter use. This is interesting because the use of alternative transcriptional start sites contributes more to transcriptome diversity than alternative splicing does in the mouse cerebellum (Pal *et al.* 2011). Because loss of *set-2* activity greatly reduces H3K4me3 in worms (Greer *et al.* 2010), it is attractive to suggest that *set-2* may play a role in regulating alternative splicing and/or alternative transcription start sites.

There are several possibilities for how alternative splicing and use of alternative promoters may affect dendrite development. The simplest possibility is that one or more specific alternatively spliced products are important for dendrite development and that defects in alternative splicing reveal defects in dendrite morphology (Olesnicky *et al.* 2014). Support for this comes from the well-documented prevalence of transcriptome diversity in the nervous system, which is thought to be important for contributing to cellular complexity (Yeo *et al.* 2004; Lipscombe 2005; Li *et al.* 2007; Norris and Calarco 2012). For example, alternative splicing of the mammalian *Bcl11A* transcriptional factor-encoding gene produces a long form that negatively regulates dendrite outgrowth and a short form that antagonizes the long form and thus promotes dendrite outgrowth (Kuo *et al.* 2009).

An alternative possibility is that some splicing occurs in the dendrites rather than in the nucleus. Several mammalian splicing components have been shown to exit the nucleus and retain function in the dendroplasm, perhaps to provide localized mature mRNAs for localized translation of specific transcripts (Glanzer *et al.* 2005; Bell *et al.* 2008). However, all of the putative splicing regulators in this study show a tight nuclear localization as observed with RBP::GFP fusion proteins (Figure 5). Because each of these fusion proteins rescues the mutant dendrite defects (except DDX-17, which was not amenable to testing), it suggests that the strong nuclear localization reflects normal activity (Figure 7). Thus, if any of these factors are involved in activities within dendrites, they must be present in concentrations that are below the level of detection by confocal microscopy. Furthermore, cytoplasmic splicing has thus far not been described in *C. elegans*.

Proteins associated with splicing also may be important for mRNA localization and/or translational regulation. For example, mammalian Mbnl proteins, which are homologs of MBL-1, are important for targeting hundreds of mRNAs to membranes where they are translated (Adereth *et al.* 2005; Osborne *et al.* 2009; Du *et al.* 2010; Masuda *et al.* 2012; Wang *et al.* 2012). Again, we do not favor this model for how the splice factors identified in this study affect dendrite development because their localization is restricted to the nucleus (Figure 5). However, the localization of mRNAs can be influenced by RBPs that never leave the nucleus. For example, localization of *ASH1* mRNA in yeast requires the function of Loc1p, an RBP that is exclusively nuclear (Long *et al.* 2001). Loc1p is required for other RBPs to assemble onto *ASH1* mRNA, which then remove Loc1p, and guide its localization (Niedner *et al.* 2013). Similarly, ZBP2 is a predominantly nuclear RBP that is required for beta-actin mRNA localization in fibroblasts and neurons (Gu *et al.* 2002). ZBP2 facilitates the binding of ZBP1 to beta-actin mRNAs, which then escorts the mRNA and represses translation (Pan *et al.* 2007). Thus, there is precedent that nuclear RBPs, including at least one implicated in mRNA splicing, can affect mRNA localization without leaving the nucleus.

Knockdown of several RBP genes encoding splicing factors in *Drosophila* led to excess dendrite branches in da neurons (Olesnicky *et al.* 2014). Among these were *mbl* and *x16*, both of which are implicated in alternative splicing (Vorbrüggen *et al.* 2000; Wang *et al.* 2012). However, mutations in the worm homologs of these genes, *mbl-1* and *rsp-6*, result in a decrease in the number of dendritic termini in PVDs. Although splicing is important in both species, it is possible that the target genes of alternative splicing are not conserved and that these targets regulate different aspects of dendrite morphogenesis.

### RNA processing and dendrite morphogenesis

*mtr-4* encodes a homolog of yeast Mtr4p, an RNA helicase that modulates the polyadenylation activity of the Trf4/Air2/Mtr4 polyadenylation complex. Trf4/Air2/Mtr4 polyadenylation adds short poly(A) tails to mRNAs and small noncoding RNAs (such as small nucleolar RNAs and transfer RNAs) to mark them for degradation or processing by the nuclear exosome (Liang *et al.* 1996; Jia *et al.* 2011; Schmidt and Butler 2013). Although it is difficult to speculate on how a general process such as RNA processing and degradation may lead to a specific phenotype such as dendrite morphology, at least one study has shown that exosome activity is important for nervous system development. Loss of zebrafish *EXOSC3* activity, which is required for exosome function, resulted in spinal motor neuron developmental defects and degeneration (Wan *et al.* 2012).

### Is the microRNA/RNAi pathway important for dendrite development?

We found that *dcr-1/Dicer* mutants have a reduction in the number of terminal dendrite branches. Although this might suggest that the process of dendrite morphogenesis is partly regulated by microRNAs, this is inconsistent with our finding that a null mutation in *drsh-1/Drosha* does not cause dendrite defects. One possibility is that *drsh-1* mutants, which are recovered from heterozygous mothers, are rescued from defects by maternal *drsh-1(+)*. Maternal rescue of adult stage developmental events in *drsh-1* mutants has been suggested previously (Denli *et al.* 2004). There is some evidence that microRNAs are important for dendrite morphogenesis (Bicker *et al.* 2014). For example, rat miR-134 is localized to dendrites in the hippocampus and negatively regulates the size of dendritic spines. Brain-derived neurotrophic factor positively regulates the size of dendritic spines by alleviating miR-134 repression (Schratt *et al.* 2006). The microRNA *bantam* regulates the scaling of da neuron arbors in *Drosophila*. However, *bantam* functions within the epidermis, not the da neuron (Parrish *et al.* 2009). It is unclear from our work if *dcr-1* functions in the neuron or non-cell-autonomously. Alternatively, dendrite development may be partly regulated by endogenous RNAi mechanisms, which are *dcr-1*-dependent but *drsh-1*-independent, or *dcr-1* may have additional roles that do not involve small noncoding RNAs.

### RBPs are candidates for dendrite development in humans

By comparing results from genetic screens for dendrite morphogenesis in two similar but distinct neuronal types in distantly related species, we have identified 12 RBPs that we suggest are likely to be involved in dendrite development in many other species as well. Furthermore, these 12 proteins may constitute a minimal set of evolutionarily conserved RBPs required for dendrite development across animal phyla. This is bolstered by the fact that human orthologs of these RBP genes are expressed in the human brain based on the TiGER database (Table 3; Liu *et al.* 2008). It will be interesting to see whether future functional studies in vertebrates demonstrate a role for these RBPs in dendrite development or whether studies in humans identify any of these RBP genes as mutated in neurological disorders in humans.

**Table 3:** Human orthologs of *C. elegans* RBPs implicated in PVD dendrite development

*C. elegans* RBP	OrthoDB ID	Human Ortholog	E Value	Brain Expression
CGH-1	EOG7D85W7	DDX6	1.00E-179	Expressed
CPB-3	EOG751NG9	CPEB1	5.00E-71	Expressed
DCR-1	EOG78PV82	DICER1	2.00E-167	Expressed
DDX-17	EOG7HTHGB	DDX17	2.00E-163	Expressed
	EOG7HTHGB	DDX5	5.00E-163	Expressed
LARP-5	EOG74J971	LARP4B	2.00E-32	Expressed
	EOG74J971	LARP4	1.00E-30	Expressed
MBL-1	EOG77M8PC	MBNL2	1.00E-32	Not assayed
	EOG77M8PC	MBNL3	5.00E-31	Expressed
	EOG77M8PC	MBNL1	5.00E-30	Not assayed
MTR-4	EOG7XSTCX	SKIV2L2	0.0	Expressed
RSP-3	EOG76X620	SRSF1	5.00E-56	Expressed
	EOG76X620	SRSF9	3.00E-53	Not assayed
RSP-6	EOG73NG5V	SRSF3	9.00E-23	Expressed
	EOG73NG5V	SRSF7	3.00E-22	Expressed
SET-2	EOG72JWHB	SETD1A	6.00E-65	Expressed
	EOG72JWHB	SETD1B	7.00E-60	Expressed
SUP-26	EOG71G9V3	RBMS1	4.00E-53	Expressed
	EOG71G9V3	RBMS2	8.00E-52	Expressed
	EOG71G9V3	RBMS3	9.00E-52	Expressed
Y55F3AM.3	EOG71K63D	RBM39	3.00E-95	Expressed
	EOG71K63D	RBM23	3.00E-53	Expressed

Human orthologs were identified using the Database of Orthologous Groups and are displayed with the corresponding OrthoDB IDs. *C. elegans* proteins were aligned to the human protein RefSeq database using BLASTp and E values are given. Gene expression in the brain was determined using the TiGER database (Liu *et al.* 2008). RBP, RNA-binding protein; TiGER, Tissue-specific Gene Expression and Regulation.

## 

## Supplementary Material

Supporting Information
