# Supplementary material for: Conserved RNA-Binding Proteins Required for Dendrite Morphogenesis in Caenorhabditis elegans Sensory Neurons
Source: G3 (Bethesda). 2015 Feb 10;5(4):639–53. doi: 10.1534/g3.115.017327 (PMC4390579; doi:10.1534/g3.115.017327)
Supplement: Supporting Information [file supp_5_4_639__index.html]

Conserved RNA-Binding Proteins Required for Dendrite Morphogenesis in Caenorhabditis elegans Sensory Neurons — Supporting Information 

# Conserved RNA-Binding Proteins Required for Dendrite Morphogenesis in *Caenorhabditis elegans* Sensory Neurons

## Supporting Information for Antonacci *et al.*, 2015

**Files in this Data Supplement:**

- Supporting Information - Tables S1-S2 (PDF, 145 KB)
- Table S1 - List of RBP genes screened. (PDF, 111 KB)
- Table S2 - List of sequences and sources for transgene construction. (PDF, 123 KB)
