# Supplementary material for: Conserved RNA-Binding Proteins Required for Dendrite Morphogenesis in Caenorhabditis elegans Sensory Neurons
Source: G3 (Bethesda). 2015 Feb 10;5(4):639–53. doi: 10.1534/g3.115.017327 (PMC4390579; doi:10.1534/g3.115.017327)
Supplement: Supporting Information [file supp_g3.115.017327_017327SI.pdf]

G3: Genes | Genomes | Genetics

Conserved RNA-Binding Proteins Required for Dendrite Morphogenesis in *Caenorhabditis elegans* Sensory Neurons

Simona Antonacci*, Daniel Forand#, Margaret Wolf*, Courtney Tyus*, Julia Barney*, Leah Kellogg*, Margo A. Simon*, Genevieve Kerr*, Kristen L. Wells*, Serena Younes#, Nathan T. Mortimer§, Eugenia C. Olesnicky#, Darrell J. Killian*

* Department of Molecular Biology, Colorado College, Colorado Springs, CO, 80903

Department of Biology, University of Colorado Colorado Springs, Colorado Springs, CO, 80918

§ Department of Biological Sciences, University of Denver, Denver, CO, 80208

Corresponding authors: Eugenia C. Olesnicky, Department of Biology, University of Colorado Colorado Springs, 1420 Austin Bluffs Parkway, Colorado Springs, CO 80918. Email: eolesnic@uccs.edu; Darrell J. Killian, Department of Molecular Biology, Colorado College, 14 East Cache La Poudre Street, Colorado Springs, CO 80903. Email: darrell.killian@coloradocollege.edu

DOI: 10.1534/g3.115.017327

Table S1 List of RBP genes screened

C. elegans RBP	*Drosophila* RBP	BLASTP E value	Allele	Reference	RNAi
B0336.3	Swm	3.00E-16	*gk910*	UBC	III-3A15
B0511.6	Pit	1.00E-175	*ok2948*	UBC	This study
C25A1.4	Rump	1.00E-46	none		I-5G19
C30B5.4	CG10466	2.00E-48	*gk3082*	UBC	II-4J09
C44B7.2	Sm	3.00E-69	none		This study
C46F11.4	CG6418	0.00E+00	none		III-1H08
C56G2.1	Spoon/Yu	3.00E-30	*tm3447*	NBRP	
CGH-1	Gem3	6.00E-61	*ok492*	UBC	
CPB-3	Orb	7.00E-64	*bt17*	Hasegawa *et al.* 2006	
CYN-13	Cyp33	5.00E-124	none		This study
D1037.1	Loq	6.00E-09	*ok1746*	OMRF	
DDX-17	CG10777	5.00E-160	*tm3202*	NBRP	This study
DCR-1	Dcr-1	3.00E-124	*ok247*	OMRF	
DRSH-1	Drosha	0.00E+00	*ok369*	UBC	
ETR-1	Aret/Bru	9.00E-118	*tm6221*		II-1G15
EXC-7	Ssx	1.00E-47	*ok370*		
F13E9.1	CG5439	2.00E-08	*tm1886*	NBRP	
F26B1.2	Bl	2.00E-39	*tm5522*	NBRP	
F57B10.8	CG32706	3.00E-11	none		I-3M23
FUST-1	CG14718	2.00E-08	*tm4439*	NBRP	
HEL-1	Hel25E	0.00E+00	*ok3698*		II-6I01
HRPF-1	CG11726	8.00E-06	*tm3406*	NBRP	
K08F4.2	Rin	3.00E-10	none		IV-5G18
LARP-5	CG11505	8.00E-33	*gk939577/463873**	Thompson *et al.* 2013	I-1H07

MBL-1	Mbl	5.00E-45	*tm1563*	NBRP	
MTR-4	L(2)35Df	0.00E+00	*ok2642*	OMRF	
NCL-1	Brat	2.00E-172	*e1942*	Frank & Roth 1998	
NOS-1	Nos	1.00E-07	*ok250*	OMRF	
PUF-9	Pum	6.00E-128	*ok1136*	UBC	
R05D11.4	CG5589	6.00E-112	none		I-4A04
R11A8.7	MASK	2.00E-154	*tm5136*	NBRP	
RNP-3	Snf	5.00E-70	*ok1424*	OMRF	
RPS-3	RpS3	6.00E-124	none		III-3C02
RSP-3	SF2	4.00E-59	*ok2927*	UBC	
RSP-6	X16	3.00E-22	*ok798*	UBC	
RSP-7	Srp54	8.00E-45	*ok2079*	UBC	II-7O08
SAP-49	CG11454	5.00E-10	none		II-5N14
SET-2	Set1	4.00E-68	*n4589*	Andersen & Horvitz 2007	
SQD-1	Sqd	3.00E-41	*ok1582*	UBC	
STAU-1	Stau	1.00E-42	*tm2266*	NBRP	
SUP-26	Shep	6.00E-52	*gk426*	UBC	
SYM-2	Glo	3.00E-32	*mn617*	Yochem *et al.* 2004	
T08B2.5	CG4887	2.00E-23	*gk721*	UBC	I-2P02
T28D6.4	Mib2	7.00E-23	*tm926*	NBRP	
TIAR-1	CG34354	1.00E-77	*tm361*	NBRP	
UAF-2	U2AF38	9.00E-90	*gk3159*	UBC	IV-8I21
W04D2.6	CG4119	8.00E-28	*tm2681*	NBRP	
WDFY-2	CG5168	2.00E-98	*tm3806*	NBRP	
Y23H5B.6	CG5800	1.00E-172	none		This study
Y55F3AM.3	CG11266	1.00E-97	*gk454899***	Thompson *et al.* 2013	IV-8L05
Y55F3BR.1	Ddx1	0.00E+00	none		IV-1G20
ZC190.4	Smg	7.00E-17	none		This study

ZC434.3	CG9107	1.00E-11	none		This study
ZK686.2	Dbp73D	4.00E-59	*tm5978*	NBRP	

C. elegans RBPs are the best homologs of *Drosophila* RBPs reported by Olesnicky *et al.* (2014) by BLASTp search (E values are given). Sources of alleles are indicated above. Abbreviations: (NBRP) the Mitani Lab through the *C. elegan*s National Bioresource Project of Japan; (UBC) *C. elegan*s Reverse Genetics Core Facility at the University of British Columbia, (OMRF) *C. elegan*s Reverse Genetics Core Facility at the Oklahoma Medical Research Foundation. UBC and OMRF are part of the international *C. elegans* Gene Knockout Consortium. RNAi clones (clone location given) are from the Ahringer library (Geneservice LTD; Fraser *et al*. 2000; Kamath *et al.* 2003) or were constructed for this study (plasmid construction described in Table S2).

**gk939577/gk463873* results in a conceptual translation of the first 436 amino acids unchanged followed by 3 missense changes and a stop codon. There are 308 codons after the stop. The LA domain and the LARP4/5-like domain are within the first 436 amino acids.

***gk454899* is a single missense change R251C in a residue conserved in flies, fish, and mammals. The residue does not fall within anRNA recognition motif (RRM) but falls in a region between two RRMs.

Table S2 List of sequences and sources for transgene construction

A. Primer sequences for GFP transcriptional fusions

Regulatory region	Forward primer (5′ to 3′)	Reverse primer (5′ to 3′)
cgh-1	*Sph*I - tatcacagatttctggttgaatatgagg	*Kpn*I - tttccgatgtcgtagtaggtttgatttc
cpb-3	*Sph*I - accggaggaagcttggtgaagcaatg	*Kpn*I - ggtgaatgcttttatcaacggc
dcr-1	*Sph*I - aggattaaaatcttgaacatattgcccg	*Kpn*I - tattggtctgaaaacagggaaaattgat
ddx-17	*att*B1 - gtatttcgtgacgtcactgcg	*att*B2 - tatcctctgtctcccattgtc
larp-5	*att*B1 - tccactcttccattacgttcgggc	*att*B2 - ctccgaaaagggacgtcgacgccg
mbl-1	*att*B1 - tttgggattacttcggtgctc	*att*B2 - ccgatcgtgcgcattgtcc
mtr-4	*Sph*I - tcgccacgaaaccacaccaaacggcgag	*Kpn*I - ttagtctgaaaaatgtgccacaataaaa
rsp-3	*att*B1 - gccctatcttaaatggccgg	*att*B2 - gtttagtacactgaaaatgaag
rsp-6	*att*B1 - gtgtaaatgtgatgcattcgag	*att*B2 - actgaaaaatcaaattaatttatg
set-2	*att*B1 - tgaatagcaaacttcatatcc	*att*B2 - ttcccatcaattatttattgatgc
Y55F3AM.3	*att*B1 - tcaaattgatgtttctattcgc	*att*B2 - ggagctgaccccgcttcaaa

For *sup-26,* an existing transgene, *smIs259[P$_{sup\text{-}26}$::sup-26 cDNA::GFP],* was used (Mapes *et al.* 2010).

B. Primer sequences for GFP translational fusions to GFP

cDNA (source)	Forward primer (5′ to 3′)	Reverse primer (5′ to 3′)
cgh-1 (yk85e1, yk1658a03)	*att*B1 - atgagtggagcggagcaacaacag	*att*B2 - agcagtggtctcatcggcggc
cpb-3 (this study)	*att*B1 - atgaacctgaatgaccgagtggaag	*att*B2 - accggaggaagcttggtgaagcaatg
ddx-17 (this study)	*att*B1 - atgggagacagaggatacggagg	*att*B2 - ccatcggccacctccaccgctgc
larp-5 (yk1291c05, yk400c5)	*att*B1 - atggacacgttcgagctcgactcg	*attB2* - cctgaacgtcggtttttgtggcgg
mbl-1 (this study)	*att*B1 - atgttcgacgaaaacagtaatgccg	*att*B2 - gaatggtggtggctgcatgtac
mtr-4 (yk506b7, yk430a11)	*att*B1 - atggccgatcttttcgacgaattc	*att*B2 - tagatagagagatgcagcgaaaac
rsp-3 (yk1100h10, yk1195c10)	*att*B1 - atgccacgcggcggctcagagg	*att*B2 - ttgtggagatggtgagcgagatgg

rsp-6 (this study)	*att*B1 - atggacgccaaggtgtacgtcg	*att*B2 - gtgcggagaagcagaacggctgc
set-2 (yk21g8, yk320e10)	*att*B1 - atgtccacacatgatatgaacc	*att*B2 - attaagatatccacgacacgtc
sup-26 (D. Xue, J. Mapes)	*att*B1 - atgaacgcatcttcggctccac	*att*B2 - ttgtggattcatcggctggaaatac
Y55F3AM.3 (this study)	*att*B1 - atgaccgacggatcgatgg	*att*B2 - atatctgccatatccaccatattg

C. Primer sequences for RNAi vectors

RNAi target	Forward primer (5′ to 3′)	Reverse primer (5′ to 3′)
B0511.6	*att*B1 - gcattgacgtcaagaagaaggtgc	*att*B2 - ccacccatcactaatccatacg
C44B7.2	*att*B1 - atgcaaggaagaggcggatatcatcacg	*att*B2 - atctggaagagtgaagtctcgttgatc
cyn-13	*att*B1 - aatttccagctccgccacacaatgcccg	*att*B2 - taagtctccatagcctgcggaagcggc
ddx-17	*att*B1 - tggtctgccgagaacctta	*att*B2 - caattcacgagttggaagca
rps-3	*att*B1 - cgtgaccaagaagaagaaggccg	*att*B2 - gatgtagtcgttgactgggtgtcc
Y23H5B.6	*att*B1 - tatgcctcacacaaacggaaaaggcggcg	*att*B2 - atttgtgcaaactctgcacaaatccttg
ZC190.4	*att*B1 - tatcttggatactggctgaaaaagttgcg	*att*B2 - tacacaactgggtatgatgaagggcac
ZC434.3	*att*B1 - tatgcgtctcgtgaaaccactgatagtgg	*att*B2 - ctagttatttctgagttgtcttcgaaaatc

Genomic fragments PCR-amplified with primers sequences given were cloned into pDONR221 with a BP reaction and then cloned into a double-T7 plasmid with a Gateway entry site (modified from pPD129.36) using an LR reaction (Invitrogen).
