# Supplementary material for: Conserved RNA-Binding Proteins Required for Dendrite Morphogenesis in Caenorhabditis elegans Sensory Neurons
Source: G3 (Bethesda). 2015 Feb 10;5(4):639–53. doi: 10.1534/g3.115.017327 (PMC4390579; doi:10.1534/g3.115.017327)
Supplement: Supporting Information [file supp_g3.115.017327_TableS1.pdf]

Table S1 List of RBP genes screened

C. elegans RBP	*Drosophila* RBP	BLASTP E value	Allele	Reference	RNAi
B0336.3	Swm	3.00E-16	*gk910*	UBC	III-3A15
B0511.6	Pit	1.00E-175	*ok2948*	UBC	This study
C25A1.4	Rump	1.00E-46	none		I-5G19
C30B5.4	CG10466	2.00E-48	*gk3082*	UBC	II-4J09
C44B7.2	Sm	3.00E-69	none		This study
C46F11.4	CG6418	0.00E+00	none		III-1H08
C56G2.1	Spoon/Yu	3.00E-30	*tm3447*	NBRP	
CGH-1	Gem3	6.00E-61	*ok492*	UBC	
CPB-3	Orb	7.00E-64	*bt17*	Hasegawa *et al.* 2006	
CYN-13	Cyp33	5.00E-124	none		This study
D1037.1	Loq	6.00E-09	*ok1746*	OMRF	
DDX-17	CG10777	5.00E-160	*tm3202*	NBRP	This study
DCR-1	Dcr-1	3.00E-124	*ok247*	OMRF	
DRSH-1	Drosha	0.00E+00	*ok369*	UBC	
ETR-1	Aret/Bru	9.00E-118	*tm6221*		II-1G15
EXC-7	Ssx	1.00E-47	*ok370*		
F13E9.1	CG5439	2.00E-08	*tm1886*	NBRP	
F26B1.2	Bl	2.00E-39	*tm5522*	NBRP	
F57B10.8	CG32706	3.00E-11	none		I-3M23
FUST-1	CG14718	2.00E-08	*tm4439*	NBRP	
HEL-1	Hel25E	0.00E+00	*ok3698*		II-6I01
HRPF-1	CG11726	8.00E-06	*tm3406*	NBRP	
K08F4.2	Rin	3.00E-10	none		IV-5G18
LARP-5	CG11505	8.00E-33	*gk939577/463873**	Thompson *et al.* 2013	I-1H07

MBL-1	Mbl	5.00E-45	*tm1563*	NBRP	
MTR-4	L(2)35Df	0.00E+00	*ok2642*	OMRF	
NCL-1	Brat	2.00E-172	*e1942*	Frank & Roth 1998	
NOS-1	Nos	1.00E-07	*ok250*	OMRF	
PUF-9	Pum	6.00E-128	*ok1136*	UBC	
R05D11.4	CG5589	6.00E-112	none		I-4A04
R11A8.7	MASK	2.00E-154	*tm5136*	NBRP	
RNP-3	Snf	5.00E-70	*ok1424*	OMRF	
RPS-3	RpS3	6.00E-124	none		III-3C02
RSP-3	SF2	4.00E-59	*ok2927*	UBC	
RSP-6	X16	3.00E-22	*ok798*	UBC	
RSP-7	Srp54	8.00E-45	*ok2079*	UBC	II-7O08
SAP-49	CG11454	5.00E-10	none		II-5N14
SET-2	Set1	4.00E-68	*n4589*	Andersen & Horvitz 2007	
SQD-1	Sqd	3.00E-41	*ok1582*	UBC	
STAU-1	Stau	1.00E-42	*tm2266*	NBRP	
SUP-26	Shep	6.00E-52	*gk426*	UBC	
SYM-2	Glo	3.00E-32	*mn617*	Yochem *et al.* 2004	
T08B2.5	CG4887	2.00E-23	*gk721*	UBC	I-2P02
T28D6.4	Mib2	7.00E-23	*tm926*	NBRP	
TIAR-1	CG34354	1.00E-77	*tm361*	NBRP	
UAF-2	U2AF38	9.00E-90	*gk3159*	UBC	IV-8I21
W04D2.6	CG4119	8.00E-28	*tm2681*	NBRP	
WDFY-2	CG5168	2.00E-98	*tm3806*	NBRP	
Y23H5B.6	CG5800	1.00E-172	none		This study
Y55F3AM.3	CG11266	1.00E-97	*gk454899***	Thompson *et al.* 2013	IV-8L05
Y55F3BR.1	Ddx1	0.00E+00	none		IV-1G20
ZC190.4	Smg	7.00E-17	none		This study

ZC434.3	CG9107	1.00E-11	none		This study
ZK686.2	Dbp73D	4.00E-59	*tm5978*	NBRP	

C. elegans RBPs are the best homologs of *Drosophila* RBPs reported by Olesnicky *et al.* (2014) by BLASTp search (E values are given). Sources of alleles are indicated above. Abbreviations: (NBRP) the Mitani Lab through the *C. elegan*s National Bioresource Project of Japan; (UBC) *C. elegan*s Reverse Genetics Core Facility at the University of British Columbia, (OMRF) *C. elegan*s Reverse Genetics Core Facility at the Oklahoma Medical Research Foundation. UBC and OMRF are part of the international *C. elegans* Gene Knockout Consortium. RNAi clones (clone location given) are from the Ahringer library (Geneservice LTD; Fraser *et al*. 2000; Kamath *et al.* 2003) or were constructed for this study (plasmid construction described in Table S2).

**gk939577/gk463873* results in a conceptual translation of the first 436 amino acids unchanged followed by 3 missense changes and a stop codon. There are 308 codons after the stop. The LA domain and the LARP4/5-like domain are within the first 436 amino acids.

***gk454899* is a single missense change R251C in a residue conserved in flies, fish, and mammals. The residue does not fall within anRNA recognition motif (RRM) but falls in a region between two RRMs.
