# Supplementary material for: Conserved RNA-Binding Proteins Required for Dendrite Morphogenesis in Caenorhabditis elegans Sensory Neurons
Source: G3 (Bethesda). 2015 Feb 10;5(4):639–53. doi: 10.1534/g3.115.017327 (PMC4390579; doi:10.1534/g3.115.017327)
Supplement: Supporting Information [file supp_g3.115.017327_TableS2.pdf]

Table S2 List of sequences and sources for transgene construction

A. Primer sequences for GFP transcriptional fusions

Regulatory region	Forward primer (5′ to 3′)	Reverse primer (5′ to 3′)
cgh-1	*Sph*I - tatcacagatttctggttgaatatgagg	*Kpn*I - tttccgatgtcgtagtaggtttgatttc
cpb-3	*Sph*I - accggaggaagcttggtgaagcaatg	*Kpn*I - ggtgaatgcttttatcaacggc
dcr-1	*Sph*I - aggattaaaatcttgaacatattgcccg	*Kpn*I - tattggtctgaaaacagggaaaattgat
ddx-17	*att*B1 - gtatttcgtgacgtcactgcg	*att*B2 - tatcctctgtctcccattgtc
larp-5	*att*B1 - tccactcttccattacgttcgggc	*att*B2 - ctccgaaaagggacgtcgacgccg
mbl-1	*att*B1 - tttgggattacttcggtgctc	*att*B2 - ccgatcgtgcgcattgtcc
mtr-4	*Sph*I - tcgccacgaaaccacaccaaacggcgag	*Kpn*I - ttagtctgaaaaatgtgccacaataaaa
rsp-3	*att*B1 - gccctatcttaaatggccgg	*att*B2 - gtttagtacactgaaaatgaag
rsp-6	*att*B1 - gtgtaaatgtgatgcattcgag	*att*B2 - actgaaaaatcaaattaatttatg
set-2	*att*B1 - tgaatagcaaacttcatatcc	*att*B2 - ttcccatcaattatttattgatgc
Y55F3AM.3	*att*B1 - tcaaattgatgtttctattcgc	*att*B2 - ggagctgaccccgcttcaaa

For *sup-26,* an existing transgene, *smIs259[P$_{sup\text{-}26}$::sup-26 cDNA::GFP],* was used (Mapes *et al.* 2010).

B. Primer sequences for GFP translational fusions to GFP

cDNA (source)	Forward primer (5′ to 3′)	Reverse primer (5′ to 3′)
cgh-1 (yk85e1, yk1658a03)	*att*B1 - atgagtggagcggagcaacaacag	*att*B2 - agcagtggtctcatcggcggc
cpb-3 (this study)	*att*B1 - atgaacctgaatgaccgagtggaag	*att*B2 - accggaggaagcttggtgaagcaatg
ddx-17 (this study)	*att*B1 - atgggagacagaggatacggagg	*att*B2 - ccatcggccacctccaccgctgc
larp-5 (yk1291c05, yk400c5)	*att*B1 - atggacacgttcgagctcgactcg	*attB2* - cctgaacgtcggtttttgtggcgg
mbl-1 (this study)	*att*B1 - atgttcgacgaaaacagtaatgccg	*att*B2 - gaatggtggtggctgcatgtac
mtr-4 (yk506b7, yk430a11)	*att*B1 - atggccgatcttttcgacgaattc	*att*B2 - tagatagagagatgcagcgaaaac
rsp-3 (yk1100h10, yk1195c10)	*att*B1 - atgccacgcggcggctcagagg	*att*B2 - ttgtggagatggtgagcgagatgg

rsp-6 (this study)	*att*B1 - atggacgccaaggtgtacgtcg	*att*B2 - gtgcggagaagcagaacggctgc
set-2 (yk21g8, yk320e10)	*att*B1 - atgtccacacatgatatgaacc	*att*B2 - attaagatatccacgacacgtc
sup-26 (D. Xue, J. Mapes)	*att*B1 - atgaacgcatcttcggctccac	*att*B2 - ttgtggattcatcggctggaaatac
Y55F3AM.3 (this study)	*att*B1 - atgaccgacggatcgatgg	*att*B2 - atatctgccatatccaccatattg

C. Primer sequences for RNAi vectors

RNAi target	Forward primer (5′ to 3′)	Reverse primer (5′ to 3′)
B0511.6	*att*B1 - gcattgacgtcaagaagaaggtgc	*att*B2 - ccacccatcactaatccatacg
C44B7.2	*att*B1 - atgcaaggaagaggcggatatcatcacg	*att*B2 - atctggaagagtgaagtctcgttgatc
cyn-13	*att*B1 - aatttccagctccgccacacaatgcccg	*att*B2 - taagtctccatagcctgcggaagcggc
ddx-17	*att*B1 - tggtctgccgagaacctta	*att*B2 - caattcacgagttggaagca
rps-3	*att*B1 - cgtgaccaagaagaagaaggccg	*att*B2 - gatgtagtcgttgactgggtgtcc
Y23H5B.6	*att*B1 - tatgcctcacacaaacggaaaaggcggcg	*att*B2 - atttgtgcaaactctgcacaaatccttg
ZC190.4	*att*B1 - tatcttggatactggctgaaaaagttgcg	*att*B2 - tacacaactgggtatgatgaagggcac
ZC434.3	*att*B1 - tatgcgtctcgtgaaaccactgatagtgg	*att*B2 - ctagttatttctgagttgtcttcgaaaatc

Genomic fragments PCR-amplified with primers sequences given were cloned into pDONR221 with a BP reaction and then cloned into a double-T7 plasmid with a Gateway entry site (modified from pPD129.36) using an LR reaction (Invitrogen).
